# Variation in allele frequencies in benzimidazole resistant and susceptible isolates of *Haemonchus contortus* during patent infection in lambs

**DOI:** 10.1038/s41598-023-28168-0

**Published:** 2023-01-23

**Authors:** Michaela Urda Dolinská, Alžbeta Königová, Georg von Samson Himmelstjerna, Marián Várady

**Affiliations:** 1 Institute of Parasitology of the Slovak Academy of Sciences, Hlinkova 3, 040 01 Košice, Slovakia; 2 Institute for Parasitology and Tropical Veterinary Medicine, Freie Universität Berlin, Robert-Von-Ostertag-Str. 7-13, 14163 Berlin, Germany

**Keywords:** Parasitology, Molecular biology

## Abstract

We evaluated the variation in the frequency of benzimidazole (BZ) resistance-associated alleles at codons 200, 167 and 368 (F200Y, F167Y, V368L) of the β-tubulin isotype 1 gene during the patent period in isolates of *Haemonchus contortus* susceptible and resistant to BZ using pyrosequencing. Four lambs 5–6 months old were infected with 5000–6000 infective third-stage larvae (L3) of the susceptible MHco1 and the multi-resistant MHco4 isolates, respectively. Faecal samples were collected 28-times during 20–90 days post-infection (dpi). Coprocultures were subsequently prepared to produce L3 for genotyping. The frequency of the resistant allele (TAC) at codon 200 in MHco1 was lowest at 43 and 76 dpi with at each time point 0% and highest at 36 dpi with 10.85%, with a mean of 6.47% ± 2.39 and a coefficient of variation of 37.01%. The frequency of the TAC at codon 200 in MHco4 was lowest at 76 dpi with 25.6% and highest at 90 dpi with 49.25%, with a mean of 35.7% ± 4.42 and a coefficient of variation of 12.39%. No resistance alleles were detected in MHco1 at either codon 167 or 368. For MHco4 isolate, resistance alleles were detected only on codon 167 with a mean of 8.00% ± 4.83 and a mean coefficient of variation of 60.40%. Our results demonstrate the considerable variation in the frequency of resistant alleles in the susceptible and resistant isolates during the patent period. This variation should be considered when testing for the presence of BZ resistance in populations of gastrointestinal parasites, especially those with a low frequency of TAC.

## Introduction

*Haemonchus contortus*, a blood-feeding endoparasite, is responsible for economic losses in livestock production. The main symptom of haemonchosis, anaemia, is due to the haematophagous feeding of the larval and adult stages of this parasite in the abomasum^1^. This nematode parasite is more common in sheep than cattle^2^. Benzimidazoles (BZs) are the most commonly used anthelmintics in veterinary medicine. The extensive use of these drugs has led to the development of BZ resistance. The spread of BZ resistance is a major problem in intensive small-ruminant production around the world, leading to major health problems and great economic losses^3,4^.

Monitoring the effectiveness of drugs is necessary to prevent the spread of anthelmintic resistance (AR). Several diagnostic methods are used for detecting AR: in vivo, in vitro, and molecular tests. In vivo and in vitro tests are also most commonly used to test the effect of anthelmintics, but each has specific limitations and disadvantages. In general, these tests cannot detect resistance if only 25% of the test population is resistant^5^, which can be overcome by using more sensitive parameters, such as discriminating doses, ED_99_/LD_99_ (concentration of anthelmintic required to kill 99% of the eggs/concentration of anthelmintic where development to the L3 stage is inhibited by 99%) and the minimum inhibitory concentration at which tests can detect low levels (< 5%) of resistance in a population^6^. Variations of the output data (ED_50_) during the patent period have also been recorded for *H. contortus* and *T. circumcincta*^7–9^. In studies, with *Haemonchus contortus* the ED_50_ values during the patency decreased/increased several times below/higher than the threshold for susceptibility/resistance 0.1 mg ml^−1^ of thiabendazole, suggesting that the in vitro egg hatch test is unable to detect resistance/susceptibility and thus the isolate might be incorrectly assigned by this criterion as susceptible/resistant^6,9^.

Molecular testing of BZ resistance, based on a comparatively well-advanced understanding of BZ resistance mechanisms, has been found to be considerably more sensitive than phenotypic testing^10^. BZs interact with β-tubulin, a protein necessary together with α–tubulin, as well as other factors for the formation of microtubules (tubular structures forming the cytoskeletal system of a cell) in the cells of parasites and disturb the balance of microtubule formation. The microtubules depolymerise, and the cells are therefore unable to absorb nutrients, with the subsequent reduction of glycogen levels and starvation of the parasite^11^. The substitution of a phenylalanine (TTC) with a tyrosine (TAC) at positions 200 (F200Y)^12^, 167 (F167Y)^13^ and V368L^14^ of the β-tubulin isotype 1 gene is the main genetic mechanism of BZ resistance in gastrointestinal parasites of domestic animals. Ghisi et al*.*^15^ identified a third point mutation in β-tubulin at position 198 (E198A), a transversion from glutamate (GAA) in susceptible populations to alanine (GCA) and a range of additional polymorphisms at this latter position has recently been documented in resistant field populations^16–20^. However, the mutation at codon 200 is considered as the most relevant polymorphism associated with BZ resistance at least in the European context^10,13,21–24^. Pyrosequencing protocols have been used for the quantification of the BZ resistance allele frequencies in DNA extracted from pooled larval samples^10^.

We designed this study to determine if pyrosequencing analysis of the F200Y, F167Y and V368L polymorphism in the β-tubulin isotype 1 gene could detect variations in the frequencies of resistant alleles during the patent period in susceptible and resistant isolates of *H. contortus*.

## Results

**Figure 1:**
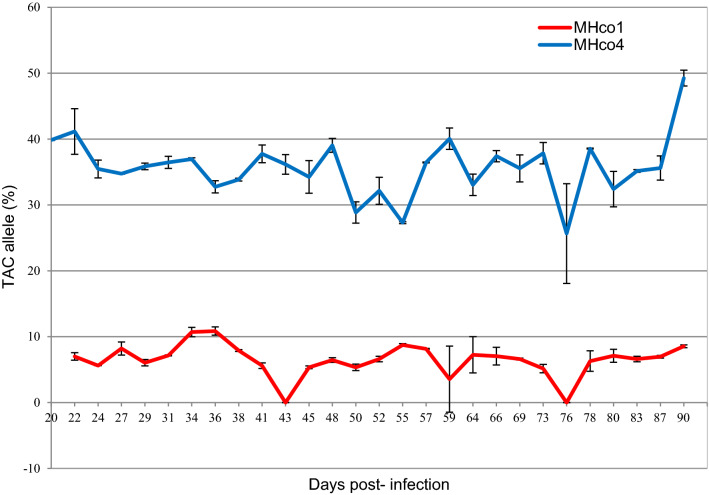
Mean ± SD of the β-tubulin benzimidazole resistance-associated allele (TAC) at codon 200 in the susceptible and resistant isolates of *H. contortus* during the course of an experimental infection (20–90 days).

Figure 1 shows means ± SD values of the frequencies of the β-tubulin codon 200 TAC in the susceptible and resistant isolates of *H. contortus* throughout the study period (Supplementary file 1). The frequency of this allele in MHco4 was lowest on day 76 with 25.6% and highest on day 90 with 49.25%, with a mean of 35.7% ± 4.42 and coefficient of variation of 12.39%. The frequency of the TAC in the MHco1 isolate was lowest on days 43 and 76 with 0% and highest on day 36 with 10.85%, with a mean of 6.47% ± 2.39 and a coefficient of variation of 37.01%. Pyrosequencing was also used to measure allele frequencies at positions 167 and 368 in both isolates on selected dpi (24, 34, 45, 55, 66, 76 and 87). We did not detect TAC at codon 368 in both isolates and at codon 167 in MHco1 isolate, while in MHco4 isolate, the mean frequency of this allele was 8.00% ± 4.83 and coefficient of variation of 60.40%. Grubbs’s test revealed one statistically significant (*p* < 0.05) outlier obtained from D90 in the MHco4 isolate at codon 200.

## Discussion

*H. contortus*, a gastrointestinal parasite in ruminants, is considered to be the most pathogenic nematode and the parasite in which resistance and multiple resistance to anthelmintics have been most frequently documented^25^. Methods for detecting AR have been gradually developed, from in vivo, to in vitro, and finally to molecular methods. The aim of this study was therefore the pyrosequencing analysis of the F200Y, F167Y and V368L polymorphism in the β-tubulin isotype 1 gene and detection of variations in the frequencies of resistant alleles during the patent period in susceptible and resistant isolates of *H. contortus*.

For example, changes in ED_50_ in the egg hatch test and in LD_50_ in the larval development test have been documented^7–9,26^. The principal question raised by these studies is whether the variation is caused by the biological properties of the isolates tested or simply by the variation of the tests. The first possibility is more likely, because both of these in vitro tests used for detecting BZ resistance are relatively sensitive and can reliably identify a small percentage (< 4%) of resistance in a population^6^. The detection of at least 10% TAC is a conservative estimate for the sensitivity of molecular techniques such as real-time PCR and pyrosequencing^10,27^. Variation in the pyrosequencing results, which we found in our study, most likely indicates the actual ratio of TAC and TTC in parasites tested on given sampling days due to the low rates of false- positive/negative results.

Our results report the frequencies of TTC and TAC, so the question remains of why our results for individual days of the patent period were so variable. This phenomenon has several possible explanations. First, whether or not the proportion of TAC and TTC alleles in L3 used for pyrosequencing represents the same proportion of alleles in L3 in the coproculture from the day after infection is questionable. We used genomic DNA isolated from a pool of approximately 5000 L3 of *H*. *contortus* from each isolate. The number of L3 from which we isolated gDNA was likely sufficient to provide a representative subsample, even though we could have used more because the fecundity of *H. contortus* females is high.

Second, we obtained the L3 from eggs produced by female parasites, so the fecundity and mortality of females may also play a role in the frequencies of TAC and TTC. This nematode is well known as a highly fecund parasite, and large populations contain a high degree of genetic variability^28^. Dinnen and Wagland^29^ reported that the fecundity of *H. contortus* in sheep ranged from 5163 to 7504 eggs per female per day. Cringoli et al*.*^30^ demonstrated that the circadian rhythm of goats did not significantly affect the faecal egg counts of gastrointestinal parasites. However, Rinaldi et al*.*^31^ found that the month of collection of faecal samples had a large effect, probably due to the mortality of the parasites. Parasite mortality in treated hosts likely depends on the initial dose of infection. Coyne et al*.*^32^ reported that the mean life span of *H. contortus* was approximately 42.1 days after infection with 500 L3 and 13.3 days after infection with 20 000 L3. Barger and LeJambre^33^, however, reported that the mean life span of *H. contortus* was 100 days without any dose-dependent infection. Under these conditions, infection with approximately 5000 L3, the shortness of our experiment (90 days), and the impossibility of reinfection, a part of the parasite population may have died**,** which could affect the representation of alleles in the remaining population and would be particularly relevant if resistant and susceptible worms differ in their survival or fecundity.

Third, the mating of sexually mature males and females may give rise to homozygous or heterozygous genotypes for resistant and susceptible genes. Eggs may thus have a variable genetic structure due to this genetic heterogeneity within the same population, which may lead to an unequal proportion of resistant alleles in the eggs. The number of heterozygous individuals in the isolates may also have played an important role: more heterozygous individuals will produce more diverse offspring. Čudeková et al*.*^34^ reported that the MHco1 and MHco4 isolates contained 10 and 15% heterozygous individuals, respectively. Such a relatively high proportion of heterozygotes could play an important role in influencing the ratio of resistant and susceptible alleles in L3.

Finally, if there are several species of gastro-intestinal nematodes in the pasture and regular reinfections occur, then the variability of resistant and susceptible genotypes can be even more unpredictable.

In conclusion, we have demonstrated considerable variation in the frequency of TAC in susceptible and resistant isolates of *H*. *contortus* during the course of infection. These changes probably represent the accurate representation of TAC in a given population rather than shortcomings in the molecular method used. These variations should be considered when testing for the presence of BZ resistance in populations of gastrointestinal nematodes, especially those with low frequencies of TAC.

## Material and methods

### Nematode populations

Two isolates of *H. contortus* were used, one susceptible and one resistant to BZs. MHco1, isolated in eastern Africa (Kenya), is susceptible to all the main classes of anthelmintics^35^. MHco4, originally isolated from the field in South Africa^36^, is resistant to BZs, rafoxanide, closantel, and ivermectin.

### Trial design

Four worm-free Improved Valachian lambs 5–6 months old were orally infected with 5000–6000 L3 of the susceptible MHco1 and the resistant MHco4 isolates of *H. contortus* (two per isolate). The animals were individually housed, and faecal samples were collected 28-times (within 20–90 days post-infection) during patency. Coprocultures were subsequently prepared for each sampling day using standard techniques^37^ to produce L3 for genotyping. Fresh L3 obtained from coprocultures were immediately stored in ethanol at room temperature till extraction of DNA.

### Pyrosequencing assays for determining β-tubulin allele frequencies

Pyrosequencing was performed according to von Samson Himmelstjerna et al.^10^. Genomic DNA was extracted from approximately 5000 *H. contortus* L3 of the susceptible and resistant isolates for each sampling day and was used as the template for PCR. The DNA was extracted using the NucleoSpin Tissue 8 kit (Macherey–Nagel, Düren, Germany) following the manufacturer’s instructions. One µl of DNA was amplified in a mixture containing 5 U of HotFire DNA polymerase (Solis Biodyne, Tartu, Estonia), 1.5 mM MgCl_2_, 80 nM dNTPs, and 200 µM forward and reverse primers. Amplification was performed with an initial denaturation and polymerase-activation step at 95 °C for 15 min, followed by 40 cycles at 95 °C for 1 min, 53 °C for 1 min, and 72 °C for 1 min, and a final elongation step at 72 °C for 10 min. We used pyrosequencing assays targeting the F200Y codon of the β-tubulin isotype-1 gene s (GenBank Accession number M76493) described by von Samson-Himmelstjerna et al*.*^10^. β-tubulin isotype 1 was amplified using the forward primer HcPy2PCR For (5′-GACGCATTCACTTGGAGGAG-3′) and a biotinylated reverse primer HcPy2PCR Rev (5′Biotin-CATAGGTTGGATTTGTGAGTT-3′). The pyrosequencing software creates a pyrogram based on the relative peak heights of the alleles, from which the frequency of each allele is then determined^10^. Each assay was performed in two independent measurements (two pyrosequencing assays per each sampling day in each isolate). ﻿Shapiro-Wilk test (*p* < 0.05) and Grubbs test have been used to test the normality and homogeneity of the data and identify potential outliers data from both isolates.

### Ethics statement

The Ethics Committee of the Institute of Parasitology of the Slovak Academy of Sciences approved animal use and experimental design under the European Community guidelines (EU Directive 2010/63/EU for animal experiments). ARRIVE (Animal Research: Reporting of In Vivo Experiments) guidelines for the use of animals were followed. The research related to animals complied with all the relevant national regulations and institutional policies for the care and use of animals. The authors confirm that they have followed EU standards for the protection of animals used for scientific purposes, and all procedures were done under European Community guidelines (EU Directive 2010/63/EU for animal experiments).

## Supplementary Information

Supplementary Information.

## Data Availability

All data generated or analysed during this study are included in this published article. The datasets used and/or analysed during the current study are available from the corresponding author upon reasonable request.
