# Supplementary material for: Variation in allele frequencies in benzimidazole resistant and susceptible isolates of Haemonchus contortus during patent infection in lambs
Source: Sci Rep. 2023 Jan 23;13:1296. doi: 10.1038/s41598-023-28168-0 (PMC9870880; doi:10.1038/s41598-023-28168-0)
Supplement: Supplementary file 1 — Supplementary Information. [file 41598_2023_28168_MOESM1_ESM.pdf]

Variation in allele frequencies in benzimidazole resistant and susceptible isolates of *Haemonchus contortus* during patent infection in lambs.

Michaela Urda Dolinská[1], Alžbeta Königová[1], Georg von Samson Himmelstjerna[2], Marián Várady[1]

codon 200	MHco1			MHco4				
**DPI**	**A**	**B**	**Mean**	**A**	**B**	**Mean**	MHco1	
20				39,9	39,8	39,85	mean	6,474074
22	6,6	7,4	7	43,6	38,7	41,15	SD	2,396346
24	5,6	5,6	5,6	36,4	34,5	35,45	CV	37,0145
27	8,9	7,5	8,2	34,7	34,8	34,75		
29	6,4	5,7	6,05	35,5	36,2	35,85		
31	7,2	7,1	7,15	35,8	37,1	36,45	MHco4	
34	10,2	11,2	10,7	36,8	37,1	36,95	mean	35,69643
36	11,3	10,4	10,85	32,1	33,4	32,75	SD	4,423757
38	8	7,8	7,9	33,7	34	33,85	CV	12,39272
41	5,9	5,3	5,6	38,7	36,8	37,75		
43	0	0	0	37,2	35,1	36,15		
45	5,5	5,2	5,35	36	32,5	34,25		
48	6,7	6,2	6,45	38,3	39,8	39,05		
50	5,7	5	5,35	27,7	30	28,85		
52	6,3	6,9	6,6	30,7	33,6	32,15		
55	8,9	8,6	8,75	27,4	27,2	27,3		
57	8,2	8,1	8,15	36,4	36,5	36,45		
59	0	7,1	3,55	41,2	38,9	40,05		
64	9,2	5,3	7,25	31,9	34,2	33,05		
66	8	6,1	7,05	36,8	38	37,4		
69	6,7	6,5	6,6	37	34,1	35,55		
73	5,6	4,7	5,15	36,7	39	37,85		
76	0	0	0	20,3	31	25,65		
78	5,2	7,4	6,3	38,5	38,6	38,55		
80	6,4	7,8	7,1	34,3	30,5	32,4		
83	6,3	6,9	6,6	35,3	35	35,15		
87	6,8	7,1	6,95	36,9	34,3	35,6		
90	8,7	8,4	8,55	50,1	48,4	49,25		

	CODON 167		
DPI	A	B	mean
24	8,5	10,4	9,45
34	8,9	7,4	8,15
45	8,7	9,5	9,1
55	0	0	0
66	10,3	9,3	9,8
76	0	10,3	5,15
87	16,4	12,4	14,4
			8,007143

Mean	8,007143
SD	4,836633
CV	60,40398

	MHco1	MHco4
DPI	Mean	Mean
20		39,85
22	7	41,15
24	5,6	35,45
27	8,2	34,75
29	6,05	35,85
31	7,15	36,45
34	10,7	36,95
36	10,85	32,75
38	7,9	33,85
41	5,6	37,75
43	0	36,15
45	5,35	34,25
48	6,45	39,05
50	5,35	28,85
52	6,6	32,15
55	8,75	27,3
57	8,15	36,45
59	3,55	40,05
64	7,25	33,05
66	7,05	37,4
69	6,6	35,55
73	5,15	37,85
76	0	25,65
78	6,3	38,55
80	7,1	32,4
83	6,6	35,15
87	6,95	35,6
90	8,55	49,25

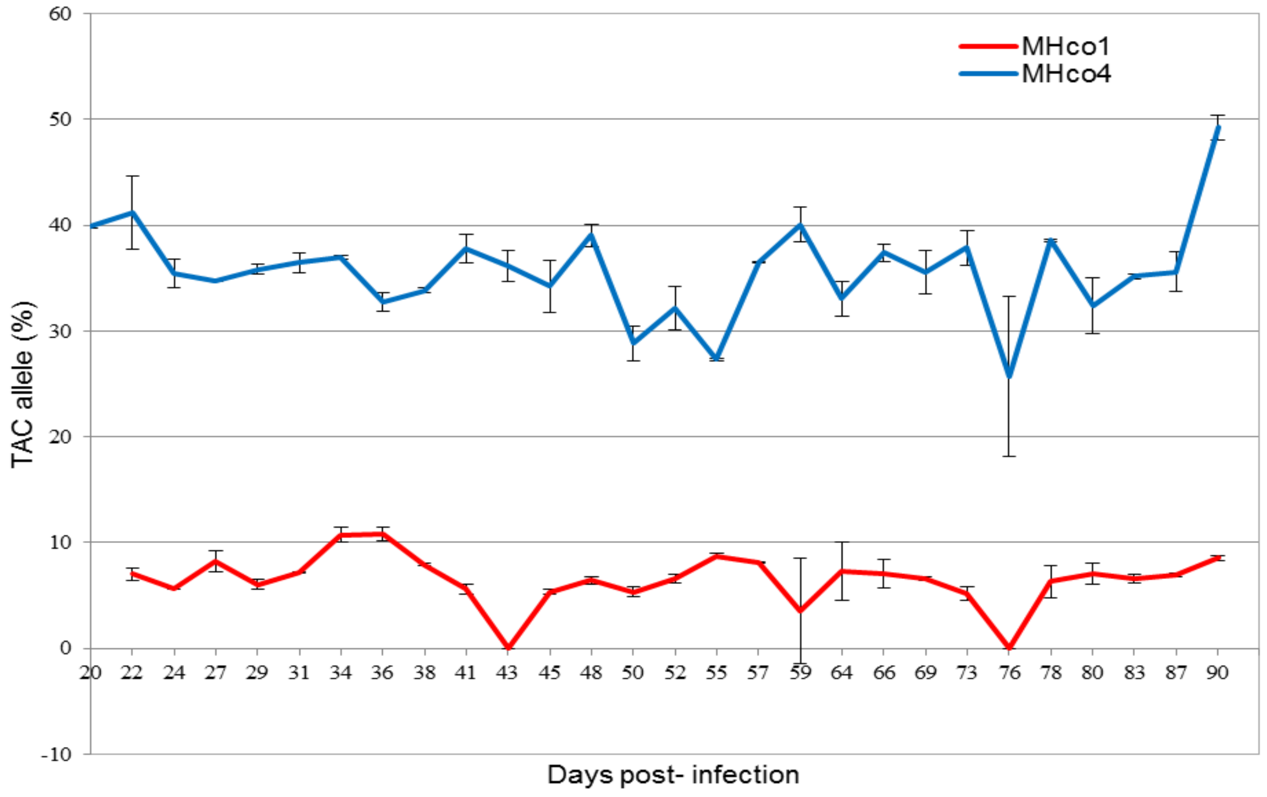

MHco1
MHco4
TAC allele (%)
60
50
40
30
20
10
0
-10
20 22 24 27 29 31 34 36 38 41 43 45 48 50 52 55 57 59 64 66 69 73 76 78 80 83 87 90
Days post- infection
